# Longitudinal urinary neopterin is associated with hearing threshold change over time in independent older adults

**DOI:** 10.1038/s41598-024-64648-7

**Published:** 2024-06-13

**Authors:** Rachel L. Kidd, Akosua Agyemang-Prempeh, Alan Sanderson, Charlotte Stuart, Sumeet Mahajan, Carl A. Verschuur, Tracey A. Newman

**Affiliations:** 1 CES, Medicine, B85, University of Southampton, Southampton, SO17 1BJ UK; 2 ISVR, USAIS, FEPs, B19, University of Southampton, Southampton, SO17 1BJ UK; 3 ENT Unit, Komfo Anokye Teaching Hospital, PO Box 1934, Kumasi, Ghana; 4 Institute of Life Sciences, B85, University of Southampton Highfield, Southampton, SO17 1BJ UK

**Keywords:** Inflammaging, Chronic inflammation, Risk stratification, Age-related hearing loss, Spectrophotometry, Auditory system, Sensory processing

## Abstract

Low-grade chronic inflammation is associated with many age-related conditions. Non-invasive methods to monitor low-grade chronic inflammation may improve the management of older people at risk of poorer outcomes. This longitudinal cohort study has determined baseline inflammation using neopterin volatility in monthly urine samples of 45 independent older adults (aged 65–75 years). Measurement of neopterin, an inflammatory metabolite, enabled stratification of individuals into risk categories based on how often in a 12-month period their neopterin level was raised. Hearing was measured (pure-tone audiometry) at baseline, 1 year and 3 years of the study. Results show that those in the highest risk category (neopterin raised greater than 50% of the time) saw greater deterioration, particularly in high-frequency, hearing. A one-way Welch’s ANOVA showed a significant difference between the risk categories for change in high-frequency hearing (W (3, 19.6) = 9.164, *p* = 0.0005). Despite the study size and duration individuals in the highest risk category were more than twice as likely to have an additional age-related morbidity than those in the lowest risk category. We conclude that volatility of neopterin in urine may enable stratification of those at greatest risk of progression of hearing loss.

## Introduction

Age related hearing loss (ARHL) is predicted to be one of the top 10 disease burdens globally by 2030, above cataracts and diabetes^1^. Unlike other chronic conditions of similar prevalence, ARHL lacks any biomedical treatment or preventative measures. Current management for hearing loss is the use of amplification in the form hearing aids or, in more severe cases, direct stimulation of the hearing system using surgically implanted prostheses such as cochlear implants. The ability to stratify people with hearing loss by risk of progession of hearing loss may support more informed clinical trials and opportunity for better individual management.

Multiple pathways and underlying conditions contribute to the biological mechanisms of AHRL^2^. No two individuals experience the same onset and progression of hearing loss. As with other age-related conditions an association has been shown between elevated inflammatory biomarkers and poorer hearing in older people^3–8^. ARHL is strongly associated with other age-related conditions that have an inflammatory element, such as diabetes and cardiovascular disease^9–13^.

Life expectancy is increasing due to advancements in medical care, improved environments, and better nutrition. There is growth in the number of elderly individuals in nearly every developed nation in the world^14^. Despite more years lived, many older people have multiple age-associated conditions in the last two decades of life often with a marked loss of quality of life and independence^15–18^. Many age-related conditions share common underlying biology; therefore, interventions targeting the molecular processes of ageing could delay both the onset and progression of age-related conditions, allowing individuals to live free of chronic disability and disease for longer^19^.

We and others have previously hypothesized that inflammaging^20,21^, the systemic and sterile low-grade chronic inflammation that occurs in many people as they age^22^ is associated with hearing loss. Multiple factors contribute to the systemic inflammatory state resulting in an imbalance of pro- and/or anti-inflammatory mediators^23^. Chronic low-grade inflammation is an accepted pathogenic factor in the development of age-related diseases such as cancer^23^, cardiovascular disease^24^, type II diabetes^25^ and hearing loss^3–6,26^, and is also a predictor of frailty^27,28^.

Studies into the effect of inflammation on hearing loss have measured inflammatory markers in blood, typically at single time points either once or years apart. Collecting longitudinal data of a biomarker over time would eliminate the effects of any daily variation in inflammation due to acute infection or inflammatory insult at the time of measurement^29^ enabling clearer assessment of low-grade chronic inflammation on the condition. No published study of ARHL to date has used this longitudinal approach, this may be in part due to participants and researchers being reluctant to engage in a study design that requires repeat blood sampling reliant on venepuncture.

Inflammatory status in older adults has been determined throught measurement of circulating pro-inflammatory cytokines, CRP, TNF-α, IL-1β and IL-6 and WBC count, however these studies have shown inconsistent findings for acute-phase reactants, including CRP, IL-6 and WBC^4–7^. An alternative is to monitor levels of stable inflammatory metabolites such as neopterin which is induced by cytokines, and that can be detected and measured in urine^30^. Human monocyte-derived macrophages and dendritic cells produce readily detectable amounts of neopterin following stimulation with interferon-γ reflecting immune activation status. Neopterin levels in urine correlate with serum levels, since neopterin does not undergo breakdown prior to release in urine^31^. For long-term monitoring, measurement of a biomarker in urine is preferable to methods that rely on detection in blood. Neopterin has been explored as a biomarker of inflammation in several conditions. Its benefit as a prognostic marker has been demonstrated in patients with chronic heart failure, where increased neopterin concentration was associated with heart failure progression after 12 months of standard chronic heart failure treatment^32^. The sensitivity and specificity of measuring neopterin in urine has been demonstrated in patients with Crohn’s Disease where measurement of urinary neopterin enabled active (flare-ups) and inactive periods of the disease to be distinguished^33^.

Obesity and inactivity^34^ are associated with elevated inflammatory markers. Higher serum neopterin levels were found in older people with reduced physical activity^35^. Sedentary living and physical inactivity are accelerants of the biological ageing process and predispose to chronic disease^36^. Higher physical activity has been shown to protect against many age-related diseases such as cardiovascular disease, cancers and neurodegenerative disorders^37,38^. Dietary management can also have a positive effect on reducing inflammation^39^, the CALERIE study in humans demonstrated that biomarkers of ageing can be modified by calorie restriction^40^. Rapamycin, aspirin and metformin which target core ageing processes directly, have been shown to extend lifespan in mice and other model organisms^41,42^. In randomised trials, metformin prevented the onset of diabetes, improved cardiovascular risk factors and reduced mortality^43,44^. Epidemiological studies suggest that metformin use might reduce the incidence of cancer and neurodegenerative disease^45^. Statins can reduce inflammatory biomarkers during chronic inflammatory conditions^46^ and in apparently healthy older individuals^47^. The effects of low-dose aspirin on progression of ARHL is the subject on an ongoing study^48^. Collectively these findings imply that better management of inflammation in later life may be benefical.

To the best of our knowledge, no published work has monitored inflammatory state with serial measurements to build an inflammatory profile of an individual over time. Taking repeated measurements of inflammation will account for variation that occurs with time as well as reducing confounding false positives for inflammaging with acute but quickly resolving inflammatory episodes. For the purpose of serial measurements over time, a biomarker that is present in urine such as neopterin is a suitable marker of inflammation. Such monitoring has the potential to be used as a screening tool to identify those with increased inflammation and therefore at risk of age-related conditions. The knowledge of individual risk category, may lead to better management and in turn progression of ARHL, and other conditions, over time.

## Method

### Participants

Convenience sampling was used to recruit a sample of community-dwelling independent older adults. The study was promoted via interest groups likely to contain a sample of older adults, in and around Southampton, UK. The groups that were targeted were chosen due to their ethos of active participation and engagement and are potentially indicative of a low burden of disease. Recruitment was geographically targeted to locations close to the testing site due to the number of study visits required, and a higher-than-average population of older adults in this region. A power calculation prior to the start of the study based on average change in hearing threshold with time that could be considered significant (> 10 dbHL ± 5 dbHL) over population average and test–retest reliability, determined a sample size of 42 to give 90% power an *α* of 0.05. Sixty-one people aged between 65 and 75 years were initially recruited, although sixteen individuals withdrew from the study during its duration resulting in a final sample of forty-five older adults. The main criterion for inclusion in the study was age, the age range was chosen to capture a cohort in whom inflammaging had potentially begun to occur. Exclusion criteria were (i) hearing loss that is severe or profound in severity (as thresholds may exceed audiometric limit), (ii) hearing loss caused by known otological pathway other than age-related hearing loss, (iii) inability to provide informed consent due to psychological condition e.g. dementia, and (iv) individuals with cancer, auto-immune diseases, or use of immunosuppressive medication, as all may inflate neopterin levels.

### Study design

**Figure 1:**
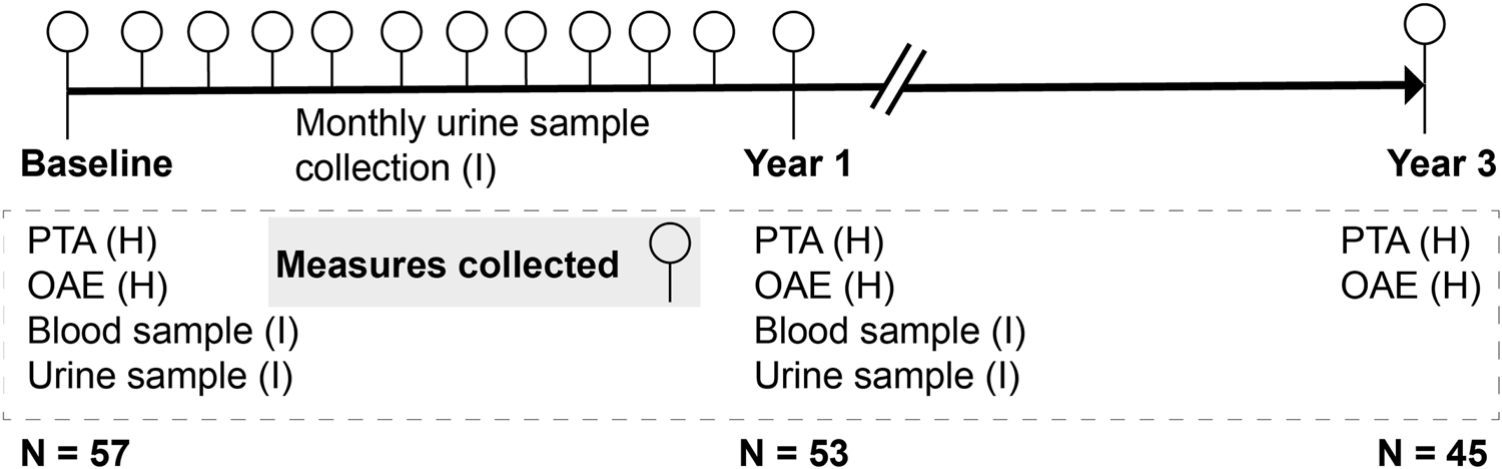
Experimental design for the study indicating the time points for the collection of hearing (H) and inflammatory (I) measures. Hearing (pure tone audiometry (PTA) and otoacoustic emissions (OAE)) measurements were collected at baseline, year 1 and year 3 of the study. Blood and urine samples were collected at baseline and year 1 of the study, for the analysis of inflammatory (I) markers including white blood cell count and cytokine levels (IL-1β, IL-6, TNF-α, IFN-γ) in the blood and levels of creatinine and neopterin in the urine. Monthly urine samples were collected within the first year of the study for neopterin analysis.

Participants attended 3 face-to-face visits at the Hearing and Balance Centre, University of Southampton. These were at baseline, end of year 1 and end of year 3. Hearing measurements were collected at each of the 3 face-to-face sessions, while inflammatory measures in blood were collected at baseline and year 1. Urine samples were collected for the months in between baseline and year 1, participants collected there samples at home and posted them to the lab using pre-prepared postal packs. Figure 1 summarises the study design.

## Methods and procedures

### Ethical approval

The research study was given ethical approval by the National Research Ethics Service, U.K., (REC: 13/SC/0507) and the University of Southampton, U.K., Ethics and Research Governance Office (reference number: 7923). All participants joined the study voluntarily. Participants gave their informed consent to join the study. It was made clear to participants that they could withdraw from the study at any point without reprisal. They could opt to have any attributable data removed from the data set, but any data that was fully anonymised at the point they withdrew would remain in the study data. Every aspect of the methodology used, and the results obtained, in the research study were performed in accordance with the regulations and requirements of the ethical apprrovals given.

### Questionnaire

A questionnaire was completed by each participant which explored demographic characteristics such as age, gender and occupation history, as well as hearing related questions such as rating hearing difficulty and questions including history of childhood ear infections, family history of hearing loss and history of noise exposure. Factors such as history of smoking were enquired about as a known factor to influence hearing. The final section asked about common chronic diseases that are associated with greater hearing loss including diabetes and cardiovascular diseases which enabled subsequent analysis of multimorbidity in relation to hearing loss and systemic inflammation.

### Pure-tone audiometry

Hearing was assessed using pure-tone audiometry (PTA) in a soundproof booth. PTA was performed using a GSI G1 Clinical Audiometer which was calibrated according to ISO-389–1:2000 Standards. PTA was performed according to the British Society of Audiology (BSA) recommended procedure^49^. Air conduction thresholds were measured at 0.25, 0.5, 1, 2, 3, 4, 6 and 8 kHz for both ears. Thresholds for bone conduction were measured at 0.5, 1, 2, 3 and 4 kHz. Commonly used audiometric averages were used to define auditory outcomes. These were defined as pure-tone average (average threshold across 500–4000 Hz), low-frequency average threshold (average threshold across 250–1000 Hz) and high-frequency threshold (average threshold across 4000–8000 Hz). A one-way repeated measures ANOVA was conducted to compare the pure-tone average threshold at baseline, year 1 and year 3.

### Measurement of blood markers

Blood was collected under aseptic conditions from the median cephalic vein in the cubital fossa into vacutainer EDTA and serum bottles. Blood in the EDTA bottle was measured for WBC immediately. Blood in the serum bottle was allowed to stand for 30–60 min to allow a clot to form. Centrifugation of the sample at 2000 rpm for 10 min at 4 °C to separate the clot so the supernatant serum could be collected. The serum was stored at − 80 °C prior to measurement of serum neopterin, IFN-γ, IL-1β, TNF-α and IL-6 levels. A HemoCue WBC and differential analyser was used to give an automated WBC and differential count (neutrophils, lymphocytes, monocytes, eosinophils and basophils). Cytokine concentrations were measured using a MSD multi-spot Pro-inflammatory panel-1 kit.

### Measurement of urinary neopterin to creatine ratio (UNCR)

Participants gave morning urine samples once a month for 12 months. The first and last samples were brought to the centre, with participants posting in their urine samples during the intervening months. Urine samples were centrifuged at 2000 rpm for 10 min and stored at − 20 °C until analysis of neopterin and creatinine levels. Urine neopterin and creatinine concentrations were measured via ultra-performance liquid chromatography-mass spectrometry (UPLC-MS)^29^. All measurements were undertaken using ACQUITY UPC interfaced with a Waters Xevo triple quadrupole (TQD) mass spectrometer equipped with an electrospray ionization probe, column oven and autosampler.

## Results

### Sample characteristics beginning to end of study

Fourty-five older adults completed the study, 11 males and 34 females, aged 65–75 years (Median 68 years) at the beginning of the study. Table 1 details some of the subject characteristics established via the questionnaires and hearing assessments. Seventeen subjects had been a smoker at some point in their lives, however eight of these had given up smoking by the age of 40 years. Four common age-related chronic diseases were enquired about in the questionnaire; diabetes, stroke and cardiovascular diseases (hypertension and coronary artery/valvular disease). Hypertension was the most common chronic disease affecting 38% of the subjects, 13% had other cardiac disease, 2% had diabetes and none of the subjects had had a stroke. 36% of subjects were being prescribed statins. The majority of the subjects (76%) had no difficulty hearing speech in a quiet environment, however, as typically seen in sensorineural hearing loss, 82% reported difficulty hearing speech in a noisy environment, and 9 had been prescribed hearing aid(s).

**Table 1:** Change in subject characteristics from baseline to end of study (questionnaire and hearing assessment data).

Characteristic	Baseline measure	Study end measure
	Number (%)	Number (%)
*Demographics*		
Age	68.98 years	72.49 years
Male	11 (24.4%)	11 (24.4%)
Female	34 (75.6%)	34 (75.6%)
*Health conditions*		
Diabetes Dx	1 (2.2%)	2 (4.4%)
Stroke	0	1 (2.2%)
Hypertension Dx	17 (37.8%)	21 (46.7%)
Heart disease Dx	6 (13.3%)	7 (15.6%)
Statins prescribed	16 (35.6%)	16 (35.6%)
One or more chronic disease Dx	18 (40.0%)	22 (48.9%)
*Hearing measures*		
Difficulty hearing speech in quiet	11 (24.4%)	15 (33.3%)
Difficulty hearing speech in noise	37 (82.2%)	37 (82.2%)
Prescribed a hearing aid	9 (20%)	13 (28.9%)
Number with normal hearing (≤ 20 dB HL)	11 (24.4%)	5 (11.1%)
Number with hearing loss (> 21–40 dB HL)	34 (75.6%)	40 (88.9%)

The same questionnaire was repeated at the end of the study. Four individuals had been diagnosed with a co-occurring chronic disease since the baseline questionnaire was completed including hypertension, heart disease and diabetes. One person reported a stroke after the baseline questionnaire. By study end, the majority of subjects (64%) still reported having no difficulty hearing speech in quiet but there was an increase in the numbers having some difficulty. The report of difficulty hearing speech in noise remained the same. Four people were prescribed a hearing aid during the period of the study.

**Figure 2:**
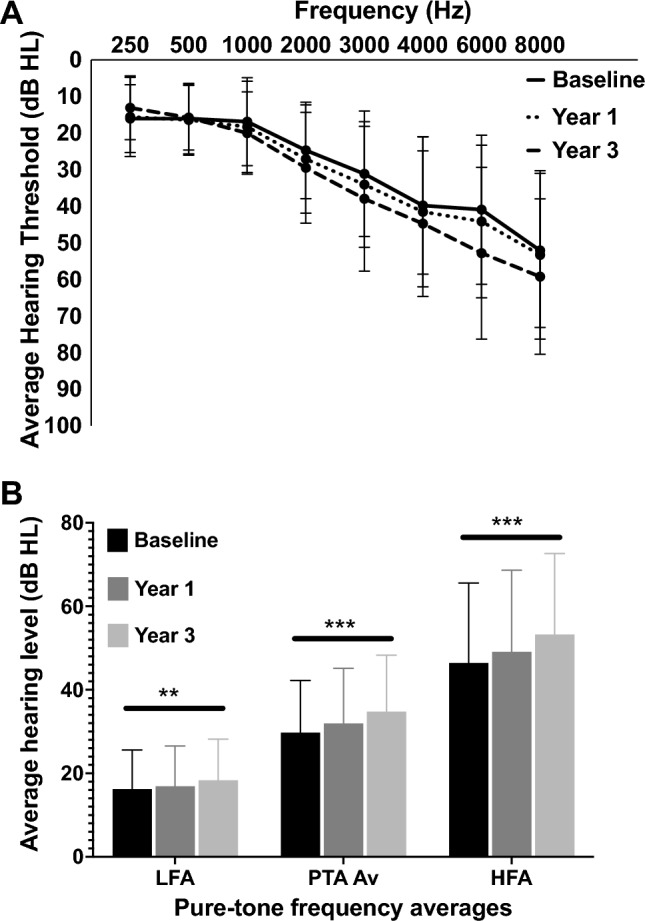
Average pure-tone audiometric thresholds for all subjects. Solid is baseline, dotted is year 1, and dashed is year 3. Worst ear air conduction thresholds used (N = 45). Error bars represent standard deviation. A) Thresholds from the worse hearing ear by air conduction across frequencies (250–8000 Hz) (N = 45). Thresholds at baseline, end of year1 and end of year 3 of the study. A deterioration in high-frequency hearing can be seen across the years. B) Average audiometric thresholds for low frequency (LFA), pure-tone (PTA Av) and high frequency (HFA) averages.

Hearing thresholds were assessed using pure tone audiometry at three time points, baseline, after 1 year and after 3 years (Fig. 2). The trend of recorded hearing loss is bilateral high-frequency sloping, which is the expected presentation of hearing loss associated with ageing^2^. Conventionally, hearing thresholds ≤ 20 dB HL are said to be within normal limits^49^. The greatest change in hearing can be seen at the higher frequencies, which is again consistent with the deterioration expected with an age-related hearing loss. From the beginning of the study to its end, the percentage of individuals with normal hearing dropped from 24 to 11% with a 7% increase in the number of individuals with both mild and moderate losses (Table 1).

There are three commonly used audiometric averages used to describe PTA data. Pure-tone average (average threshold across 500–4000 Hz), low-frequency average threshold (average threshold across 250–1000 Hz) and high-frequency threshold (average threshold across 4000–8000 Hz). These averages were calculated for the poorer hearing ear as determined by PTA Average (Fig. 2A).

A one-way repeated measures ANOVA was conducted to compare the pure-tone average threshold at baseline, year 1 and year 3 (study end) (Fig. 2B). There was a significant effect for time, Wilks’ Lambda = 0.39, F (2,43) = 34.36, *p* < 0.001, multivariate partial eta squared = 0.62. This analysis was repeated for low-frequency average and there was a significant effect for time, Wilks’ Lambda = 0.77, F (2,43) = 6.32, *p* = 0.004, multivariate partial eta squared = 0.23. The same analysis for high-frequency average produced another significant time effect, Wilks’ Lambda = 0.40, F (2,43) = 32.08, *p* < 0.001, multivariate partial eta squared = 0.60.

Both age and sex are thought to be important variables for risk of hearing loss, with older males being the most likely to have poorer hearing, In this study, the relationship between age and degree of hearing loss was investigated using Pearson product-moment correlation coefficient. This analysis showed a strong, positive correlation between age and pure-tone average thresholds, r = 0.533, n = 45, *p* < 0.001. To consider effects of sex, analyses of blood markers are presented separately for male and female participants.

### Circulating inflammatory biomarkers

**Figure 3:**
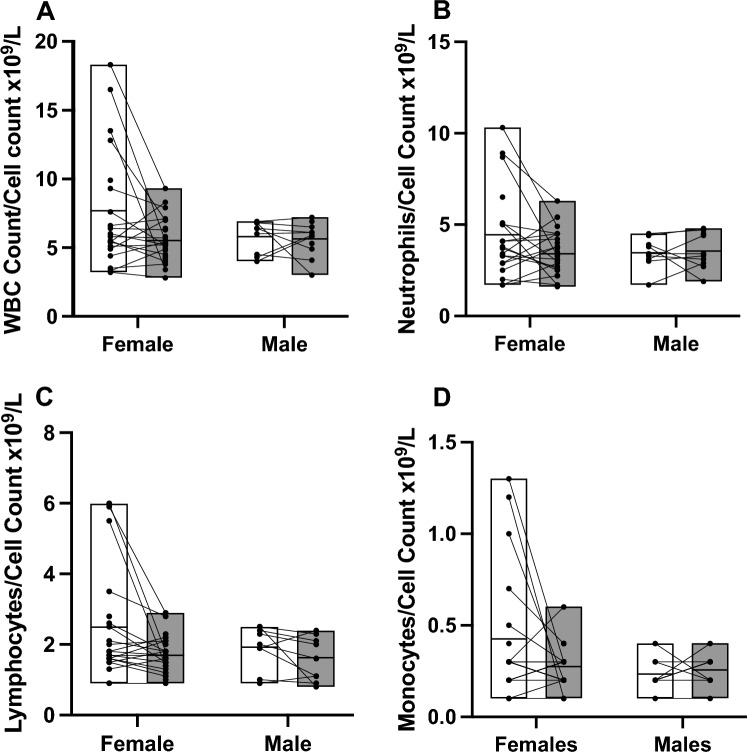
Measurements of blood markers. White is baseline data and dark grey year 1 data. Data split by sex for each marker to demonstrate no significant effect on marker due to sex. Box plot depicts mean with upper and lower limit, and symbols are the individuals with lines to show an individual’s change in measurement between the two time points. (**A**) White blood cell count. (**B**) Neutrophil count. (**C**) Lymphocyte count. (**D**) Monocyte count.

**Figure 4:**
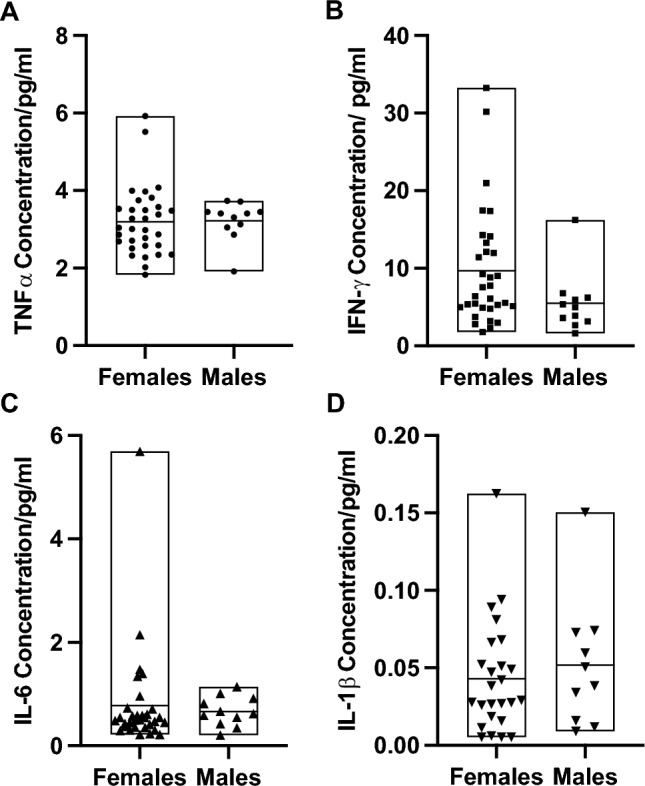
Baseline cytokine concentrations. Data split by sex for each marker to demonstrate no significant sex effect. Box plot depicts mean with upper and lower limit, and symbols are the individual values. (**A**) TNFα concentration. (**B**) Interferon -γ concentration. (**C**) Interleukin-6 concentration. (**D**) Interleukin-1β concentration.

Blood samples were collected at two time points (Baseline and after 12 months). White blood cell count (WBC), monocyte, lymphocyte and neutrophil cell counts were measured at baseline and after 1 year, the cytokines TNF-α, IFN-γ, IL-6 and IL-1β measured at baseline. Figure 3 shows that mean cell counts between the two time points are not significantly different, however, for some individuals a significant change in measure has occurred which could be due to an acute inflammatory event at either time-point, highlighting the need for longitudinal monitoring of individuals to negate false positive for inflammaging by taking single time points. Figure 4 depicts the cytokine measurements which were only measured at baseline. Variability in cytokine concentration can be seen, but this data would not be enough alone to conclude whether an increased concentration in a cytokine marker is due to inflammaging or an acute inflammatory event at the time of sampling.

### Stratification into ‘risk’ groups

Multiple measurements of neopterin for each individual over 12 months rather than measurement at a single time point, was selected to generate a variable that reflects the underlying systemic inflammatory state of an individual, or clinical variation between participants.. We used an upper limit of normal of 251 μmol/mol neopterin/creatinine based on normative values in the literature^30^ and determined what percentage of time in the 12 month period the individual had a raised (above normal) neopterin level. The cohort were stratified based on quartiles into; never raised; [neopterin levels were never raised]the sometimes raised; [levels were raised ≤ 25% of the time above physiologically normal, the often raised; [levels raised 25–50% of the time], and the considerably raised; [levels were raised > 50% of the time]. individuals who had raised neopterin levels in more than half of their samples, and that could be classified as chronically inflamed.

**Figure 5:**
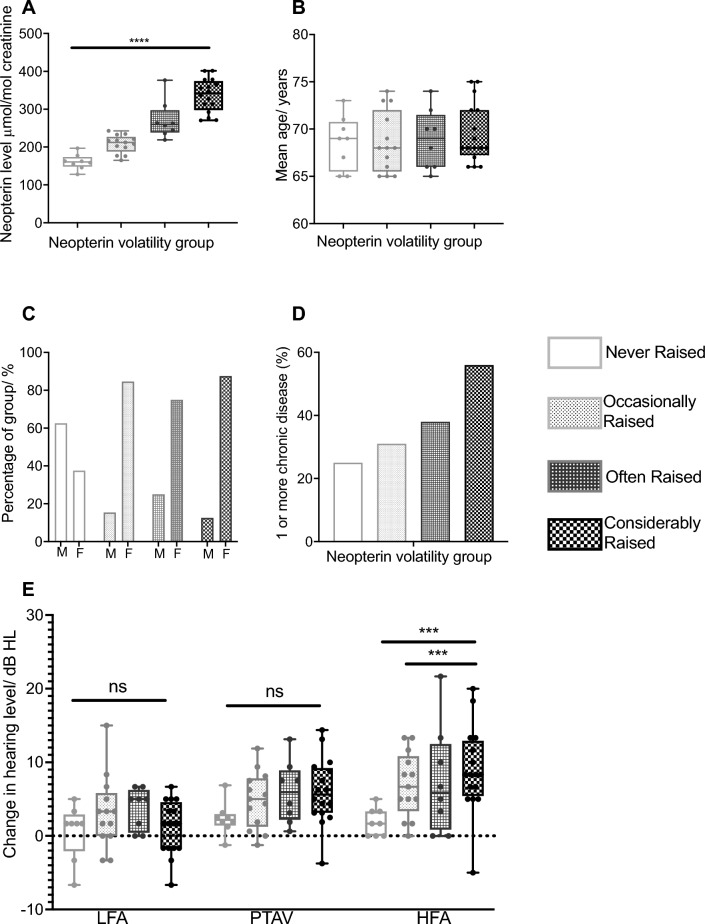
Characteristics of the stratified neopterin groups. Light grey is never raised, mid grey is sometimes raised, dark grey is often raised and black considerably raised. (**A**) Average urine neopterin level for each neopterin group, the difference between groups is significant at the *p* < 0.0001 level. (**B**) Average age in years of participants in each of the neopterin groups, there is no significant difference in age between the groups. (**C**) Percentage of male/female within each neopterin group, except the never raised group, there are considerably more females in each group than males. (**D**) Percentage of individuals within each group who are diagnosed with one or more chronic disease, those in the considerably raised group have 2.2 × greater risk of having a co-occurring age-related condition than those in the never raised group. (**E**) Mean change in audiometric threshold averages over the course of the study (Baseline to year 3) for each neopterin group. Pure-tone average (PTA), low-frequency average (LFA) and high-frequency average (HFA).

The mean neopterin level over 12 months increases with each group (Fig. 5A) as expected. There is no significant difference between the mean age of individuals within each group (Fig. 5B), therefore the increase in mean neopterin concentration between groups cannot be attributed to increasing age. When looking at the male/female ratio of each group (Fig. 5C), it is difficult to draw any firm conclusion as there were considerably more females than males in the study, however results suggest that the increase in mean neopterin concentration observed between groups is not due to sex.

The percentage of individuals within each of the stratified groups with one or more chronic age-related diseases was determined. In the never raised group, 25% subjects had at least one age-related disease, 31% of the occasionally raised group, 38% in the often raised group and 56% in the considerably raised group (Fig. 5D). The relative risk of having a co-occurring age-related disease in the considerably raised group compared to the never raised group was 2.2, therefore an individual in that groups is more than twice as likely to have a co-occurring age-related disease than those in the never raised group. After 3 years, at the end of the study, the subjects completed the questionnaire again. There was a trend that more individuals had been diagnosed with a co-occurring chronic disease, as would be expected with an ageing community^50^. An increase in percentage of the group with at least one age-related condition was seen for all groups except the considerably raised group which remained at 56%. Other work has shown that 42% of the population have one or more morbidity which is in keeping with 40% of our study cohort having one or more morbidity^50^. Multimorbidity is reported to increase rapidly with age in those over 65 years and we have observed a 7% rise in co-morbidities during the short study duration.

One of the main aims of this study was to investigate the relationship between longitudinal monitoring of neopterin as a marker of chronic inflammation and progression of age-related hearing loss. The pure-tone audiometric thresholds of every individual (within their stratified groups) were measured at baseline, end of year 1 and end of year 3. Figure 5E displays the audiometric averages for each stratified group. For change in hearing level from baseline to end of study (year 3) a Welch ANOVA was also performed for each of the audiometric averages. There was no significant difference between groups for PTA Av (W (3.000, 20.28) = 3.043 = *p* = 0.0523) or LFA (W (3.000,19.77) = 1.799, *p* = 0.1802).

Seventy-five percent of the cohort had a measurable hearing loss on testing at the start of the study compared to 89% by the end, which is typical of the prevalence of hearing loss expected in a cohort of this age^51,52^, with more people having a mild or moderate loss by the end of the study. For all three audiometric averages (Pure-tone, low-frequency and high-frequency average), a significant difference in the average hearing level (dB HL) from baseline to end of the study was observed. The greatest change to the hearing throughout the study was in the high-frequency region, which is again where the greatest deterioration due to age is expected^2,53,54^.

The Welch’s ANOVA for high-frequency average showed a significant difference between neopterin volatility groups W (3.000, 19.60) = 9.164, *p* = 0.0005. Post-hoc multiple comparisons test, showed the significant comparisons to be between the never raised and the sometimes raised groups (*p* = 0.0110) and the never raised and considerably raised groups (*p* = 0.0016).

## Discussion

This study has established an individual’s inflammatory profile, eliminating the effect of fluctuating inflammation, by taking serial monthly urinary neopterin measurements. The aim was to investigate the relationship between raised inflammatory profiles and age-related hearing loss. We wanted to assess whether inflammation is associated with poorer hearing and/or progression of hearing loss as there is growing evidence that low-grade chronic inflammation associated with ageing is a driver in age-related diseases^22^. With this in mind, control or reduction of inflammation may be a key target for therapeutic interventions in reducing the progression of age-related hearing loss.

The results demonstrate the utility of observing how often an individual’s neopterin level fluctuates above normal. There were 8 individuals in this study who never had a raised neopterin level (0% neopterin volatility) during the 12 months of the study and 3 individuals who had greater than 90% neopterin volatility during the 12 months. Only a few subjects had chronically raised inflammatory markers, this highlights that measures at single time points may not correctly categorise the subjects effectively, therefore using neopterin volatility to demonstrate the frequency of inflammatory responses, the number of times neopterin was elevated over 12 months, better indicates chronic, or inflammed, status. This is important based on the premise that the more frequent the systemic challenge, the more tissue injury caused, which has been shown in Alzheimer’s disease, where each infection or systemic challenge drives greater neuronal damage and greater decline in cognition^55^. Therefore, this study effectively demonstrates the usefulness of measuring frequency of inflammatory response to assess inflammaging (chronic inflammation), particuarly in people who do not have persistent elevated inflammatory markers.

Based on individual neopterin volatility, the cohort were stratified into categories from neopterin never-raised to considerably-raised (> 50% of the time). Those in the considerably -raised group had the highest prevalence of age-related diseases with more than twice the risk of having an age-related cardiovascular disease and type II diabetes, than those in the never-raised group, thus supporting the use of neopterin volatility as a marker of inflammation. It was then demonstrated that increased neopterin volatility, even in this relatively small cohort, was significantly associated with increased high frequency hearing loss, strongly supporting the hypothesis that inflammaging/immune activation and age-related hearing loss are linked.

The average audiogram of the individuals shows the typical configuration of hearing loss expected with ARHL- a bilateral high-frequency sloping loss^2,53,54^. Other studies have found that males have poorer hearing than females^52^, but with considerably fewer males than females in the cohort, we cannot draw any conclusion on this. Seventy-five percent of the cohort had a recognised hearing loss at the start of the study compared to 89% by the end, which is typical of the prevalence of hearing loss expected in a cohort of this age^51,52^, with more people having a mild or moderate loss by the end of the study. For all three described audiometric averages (Pure-tone average, low-frequency average and high-frequency average), a significant difference in the average hearing level (dB HL) from baseline to end of study was observed. The greatest change to the hearing throughout the study was in the high-frequency region, which is again where deterioration due to age is expected^2,53,54^.

When individuals were stratified into groups based on their neopterin volatility, a significant effect was seen in the degree of change to high-frequency hearing between groups. Particularly between the never raised group and the other groups. The mean change in high-frequency average for the never raised group was 2 dBHL and 9 dBHL for the considerably raised group, a 350% increase. Further work is needed to extend the finding to a larger cohort of older adults living in the community and to tease out underlying factors and causal relationships between disease processes related to inflammation and hearing loss itself. This could be achieved via a larger sample by establishing the co-variatoin between specific disease, pharmacological and lifestyle factors as underpinning the association, which would in turn allow better targeted intervention for hearing loss reduction. However, the present study does provide intriguing evidence that there may be a direct physiological link between unregulated chronic inflammation and one of the most prevalent and disabling age-related conditions, age-related hearing loss.

## Conclusions

Serial measurements of urine neopterin and neopterin volatility can be used to assess an individuals’ inflammatory status. High neopterin volatility is associated with increased risk and co-occurrence of chronic age-related diseases and greater progression of high-frequency hearing loss. Work to develop home testing of inflammatory markers in urine may be valuable in improving self-management.

## Data availabilty

Due to ethical restrictions these data are not publicly available. Data are however available from the corresponding author upon reasonable request and with permission of the data custodian. The data that support the findings of this study, and a link to request the data, are available from the University of Southampton repository reference doi.org/10.5258/SOTON/D2823.
